# Supplementary material for: Clinical Tick-Borne Encephalitis in a Roe Deer (Capreolus capreolus L.)
Source: Viruses. 2022 Jan 31;14(2):300. doi: 10.3390/v14020300 (PMC8875940; doi:10.3390/v14020300)
Supplement: Supplementary file 1 [file viruses-14-00300-s001.zip › DaRold_SM_final/Table S1.pdf]

Table S1: Amino acid mutations observed from the comparison of OM084948 (21RS1767), GQ266392 (AS33) and Neudoerfl in E and NS5 proteins.

Protein	AA Polyprotein	AA Protein	OM084948 (21RS1767)	GQ266392 (AS33)	U27495 (Neudoerfl)	Domain/Region
E	331	51	E	D	E	central domain (I)
	361	81	T	I	T	dimerization domain (II)
	408	128	I	I	T	central domain (I)
	447	167	V	V	I	/
	761	481	I	L	L	flavi_E_stem
NS5	2532	21	R	K	R	mRNA cap 0 and cap 1 methyltransfe domain
	2559	48	E	K	E	
	2562	58	M	M	V	
	2619	108	R	R	K	
	2764	253	R	R	K	
	2905	394	L	L	M	/
	2908	397	K	K	R	/
	2945	434	H	H	R	/
	3297	786	V	V	A	RdRp catalytic
	3366	855	K	K	R	/
